# Lower extremity muscle activity during reactive balance differs between adults with chronic traumatic brain injury and controls

**DOI:** 10.3389/fneur.2024.1432293

**Published:** 2024-09-17

**Authors:** Guilherme M. Cesar, Thad W. Buster, Judith M. Burnfield

**Affiliations:** ^1^Department of Physical Therapy, University of North Florida, Jacksonville, FL, United States; ^2^Institute for Rehabilitation Science and Engineering, Madonna Rehabilitation Hospitals, Lincoln, NE, United States; ^3^College of Medicine, Orthopedic Surgery, University of Nebraska Medical Center, Omaha, NE, United States

**Keywords:** reactive balance, electromyography, traumatic brain injury, computerized dynamic posturography, human subjects

## Abstract

**Background:**

Control of reactive balance is key to achieving safe independent walking and engagement in life activities. After traumatic brain injury (TBI), motor impairments and mobility challenges are persistent sequelae. To date, no studies have explored muscle activity of individuals with chronic TBI during a task that requires reactive control of balance.

**Objective:**

To investigate lower extremity muscle activity during a reactive balance test performed by adults with chronic severe TBI and matched controls. We hypothesized that abnormal activity of lower extremity muscles would be related with poorer reactive balance performance. Also, we performed an exploratory analysis for those with TBI investigating the impact of unilateral versus bilateral lower extremity involvement in the control of reactive balance.

**Methods:**

Ten adults with chronic severe TBI who were independent community ambulators and ten matched controls performed the computerized reactive balance test (Propriotest^®^) while lower extremity muscle activity was recorded. Electromyographic (EMG) activity was contrasted (Mann–Whitney U Test) between groups across each 10 s epoch of the 120 s test. Additionally, test scores were correlated (Spearman) with lower extremity composite EMG activity to distinguish muscle activity patterns related with reactive balance performance. Lastly, reactive balance test scores were correlated with reactive balance test scores and clinical functional measures only for the TBI group.

**Results:**

Although the TBI group exhibited greater EMG activity across the entire test compared with the control group, significant differences were not observed. Greater composite EMG activity correlated significantly with poorer reactive balance performance across most of the 10 s windows of the test.

**Conclusion:**

Greater muscle activity exhibited during the reactive balance test by individuals with chronic severe TBI compared to those without disabilities, particularly at small unexpected perturbations, highlights the greater physiologic effort required to control reactive balance even after independent ambulation is achieved.

## Introduction

Motor impairment and mobility challenges are persistent sequelae after traumatic brain injury (TBI) (1, 2). Injuries to cortical structures can disrupt the integration of information carried through sensory and motor neural pathways (3). The subsequent impaired motor control can lead to gait abnormalities (4) and place individuals at high risk of falls, limiting community integration (5–7).

While static balance (8) control contributes to standing/seating stability and function, the dynamic (9, 10) and reactive (11) components are key for engagement in life activities such as independent walking in busy community environments. Control of balance during static and dynamic conditions has been previously explored in adults with TBI. Center of pressure excursion during static standing (1) and limits of stability tests (12, 13) have provided quantifiable information regarding the impaired control of balance in contrast to individuals without disabilities. Although valuable, such kinetic information solely depicts the output of the system during static, anticipated conditions. When sensory stimuli change unpredictably, as seen during situations requiring reactive responses, appropriate motor responses to regain balance are expected to be more challenging than during anticipated conditions. To date, no studies have explored muscle activity of community-dwelling individuals with severe TBI experiencing a task requiring reactive control of balance.

This study utilized previously published data that reported differences in reactive balance control between adults with chronic severe TBI and matched controls without disabilities. The purpose of this study was to explore this difference and investigated lower extremity muscle activity during the reactive balance test performed by these adults. Given known disorganization of motor unit recruitment following brain injury (3, 14), including reduced firing rates and rate modulation (15) that can interfere with the timely contraction of muscles to counteract unexpected external perturbations, we hypothesized that abnormal activity of lower extremity muscles from those with TBI would be related with poorer performance during a motor task that requires control of reactive balance. We further performed an exploratory analysis and investigated the impact of lower extremity unilateral versus bilateral involvement on reactive balance control and functional measures.

## Materials and methods

### Participants

Our study presents a secondary analysis of a previously reported study with the same participants (11, 16, 17). Twenty adults enrolled in this Institutional Review Board approved cross-sectional study, including ten individuals (mean ± SD: 36 ± 13 years old; 172 ± 10 cm; 70 ± 16 kg) who recovered (10 ± 6 years post-injury) from severe (loss of consciousness 23 ± 23 days) TBI and ten Controls (matched by sex, age, height, and weight) without known disabilities (34 ± 13 years old; 174 ± 10 cm; 74 ± 14 kg). Even though an inclusion criterion for the TBI group was the ability to ambulate independently in the community, the TBI participants exhibited heterogeneous residual deficits such as lower extremity strength, balance, or greater unilateral *vs* bilateral involvement. Please see detailed information of our participants with TBI in Table 1.

**Table 1:** Characteristics of our participants with TBI.

Participant	Sex	Age (years)	Time Post-injury (years)	LOC (days)	BBS	DGI	Knee Extensor		Knee Flexor		Ankle Dorsiflexor		Ankle Plantarflexor		Most involved side^a^
							Left	Right	Left	Right	Left	Right	Left	Right	
							Ashworth / MMT	Ashworth / MMT	Ashworth / MMT	Ashworth / MMT	Ashworth / MMT	Ashworth / MMT	Ashworth / MMT	Ashworth / MMT	
1	M	21	4	35	55	17	0 / 5	1 / 4	0 / 5	0 / 4	0 / 5	0 / 4	1 / 5	0 / 0	Bilat
2	M	24	10	75	53	18	0 / 5	0 / 5	0 / 4	1 / 4	1 / 4	0 / 4	1 / 4	1 / 3	Bilat
3	M	24	6	14	45	15	0 / 4	0 / 4	0 / 4	0 / 4	0 / 4	0 / 4	1 / 5	1 / 5	Bilat
4	F	26	10	14	56	24	0 / 4	0 / 4	0 / 4	0 / 4	0 / 4	0 / 4	0 / 5	1 / 5	R
5	F	28	10	21	52	21	0 / 4	0 / 4	0 / 4	0 / 4	0 / 4	0 / 4	1 / 5	0 / 5	L
6	M	38	20	4	56	23	0 / 5	0 / 5	0 / 4	0 / 4	0 / 5	0 / 5	0 / 5	0 / 5	ND
7	F	40	17	11	56	24	0 / 4	0 / 4	0 / 4	0 / 4	0 / 4	0 / 4	0 / 4	0 / 4	L
8	F	46	18	49	46	19	0 / 4	0 / 4	0 / 3	0 / 4	0 / 2	0 / 4	3 / 0	0 / 3	L
9	M	53	1	7	56	24	0 / 5	0 / 5	1 / 4	0 / 4	0 / 4	0 / 5	0 / 5	0 / 5	L
10	F	55	8	2	56	24	0 / 4	0 / 4	0 / 4	0 / 4	0 / 4	0 / 4	0 / 4	0 / 4	L

a Based on scores from MMT and Ashworth Scale.

Data set is displayed from the youngest to oldest participant. BBS, Berg Balance Scale (total score 56, higher scores equal better performance); Bilat, bilateral; DGI, Dynamic Gait Index (total score 24, higher scores equal better performance); F, female; L, left; LOC, loss of consciousness; M, male; MMT, manual muscle test (total score per muscle group 5, higher scores equal better performance); ND, not determined based on MMT and Ashworth Scale; R, right.

### Instrumentation

The MA-300-10 electromyography (EMG) system used recorded lower extremity muscle activity with MA-411 surface electrodes (B&L Engineering, Santa Ana, CA, United States). Analog signals were low-pass filtered (500 Hz) using the MA-300 anti-alias filter prior to transmitting via coaxial cable to a computer where signals were digitally recorded (1,200 Hz per channel) (16). Visual 3D software (C-Motion Inc., Germantown, MD, United States) was used for subsequent signal processing.

The device PROPRIO^®^ 4000 Reactive Balance System (Perry Dynamics, Decatur, IL, United States) and the system’s computerized dynamic posturography (Propriotest^®^) were used to test reactive balance. The device is comprised of a computer-controlled platform (28-inch diameter) that progressively tilts up to 14° multi-directionally around the platform’s center point at a rate of 6°/sec to 60°/sec. The system uses ultrasonic technology (4 Hz frequency) to quantify motion of participants’ estimated center of mass. A harness (SafeLight Universal 3 M, St. Paul, MN, United States) was utilized to protect against falls.

### Experimental procedures

During the first session, participants completed the informed consent, underwent basic anthropometric evaluation (height, weight, BMI), and completed a medical history questionnaire (including details regarding their brain injury) and balance-related questionnaires explained elsewhere (11, 16, 17). Participants returned to the lab on three further occasions, scheduled at least 24 h (but no more than 72 h) apart. The data set utilized in the current study was collected during the final session, in which the biomechanical evaluation was conducted.

During the biomechanical evaluation session, each participant’s balance was first assessed clinically with two standardized tests previously validated for individuals with TBI, Berg Balance Scale (BBS) (18) and Dynamic Gait Index (DGI) (19, 20). The modified Ashworth Scale was also administered to assess muscle tone due to lesions to the central nervous system (21). Lower extremity strength measures were then collected via standardized manual muscle testing (MMT) (22, 23) by the same researcher who had over 10 years of clinical experience administering MMTs at the time of study completion. The same researcher, who had many years of experience with administering clinical tests to neurologic patient populations, administered all balance evaluations, the Ashworth Scale, and MMT in the same order to all participants. These four clinical tests were selected due to their reliability in detecting balance issues, challenging mobility tasks, muscle group weakness, and increased muscle tone in individuals with TBI. Additionally, these tests allowed for further classification of participants with TBI into sub-groups (i.e., side involvement) for our exploratory analysis.

**Figure 1:**
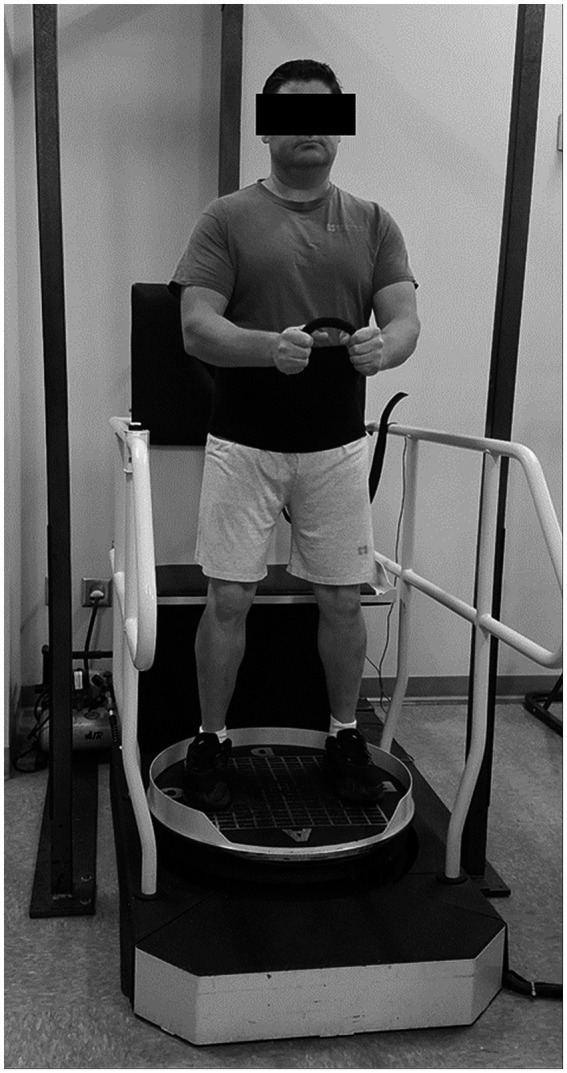
Reactive balance performance was evaluated during a computerized dynamic posturography test with the PROPRIO^®^ 4000.

Following clinical balance and strength tests, EMG surface electrodes were placed bilaterally over muscle bellies of vastus lateralis, medial hamstrings, tibialis anterior, and medial gastrocnemius. Standard electrode placement techniques were followed (24) and electrodes were secured to skin with Hypafix medical tape (BSN Medical GmbH, Quickbornstrabe 24, 20,253 Hamburg, Germany) and Coban wrap (M Corp, 3 M Center, St Paul, MN 55144-1000). After electrode placement, participants performed the reactive balance test according to manufacturer’s instructions. The system’s ultrasound transmitter was placed between the participants’ posterior superior iliac spines. On the platform, participants were instructed to look straight ahead while holding a short piece of rope between their hands (elbows flexed at 90°). The feet were positioned similarly across participants, with the outside border of each foot placed on the 4th gridline from the center of the platform while the center of each foot was placed over the medial-lateral center line (Figure 1). Participants were instructed to minimize trunk movement and, consequently, the ultrasound transmitter’s motion. No tension was applied to the fall-arresting harness during the test so balance control performance would not be impacted by an external factor. The system’s standardized test initiated with small arcs of platform motion at slow speeds followed by progressively greater amplitudes of perturbation (i.e., degrees of tilt increasing up to 14° and velocity from 6 to 60°/s). Each test lasted 120 s or until the ultrasound sensor moved greater than three inches anteriorly, posteriorly, laterally, or vertically. This test was performed three times and participants rested up to 5 min between each test.

### Data analyses

The BBS evaluates 14 functional activities, each graded on an ordinal 5-point scale (0 represents unable to perform, 4 represents perform without difficulty). The minimum and maximum BBS scores are 0 and 56, respectively, with lower scores representing worse balance performances (25). The DGI evaluates 8 walking tasks, each graded on an ordinal 4-point scale (0 represents severe impairment, 3 represents no dysfunction). The minimum and maximum DGI scores are 0 and 24, respectively, with lower scores representing severe walking and balance impairment (26). The modified Ashworth Scale measures spasticity with an ordinal scale from 0 (no increase in muscle tone) to 4 (affected segment is rigid in flexion or extension) (21). For each muscle tested for strength, the minimum and maximum MMT scores were 0 and 5, respectively, with 0 indicating individuals cannot generate any noticeable muscle contraction. The sum of all eight muscles (i.e., vastus lateralis, medial hamstrings, tibialis anterior, medial gastrocnemius, bilaterally) was used to represent lower extremity *composite muscle strength*. Thus, 40 points was the maximum composite score. Although previous research identified weaker right lower plantar flexor strength for our TBI group compared to controls (16), we selected to investigate the *composite muscle strength* since reactive standing balance responses are expected to require integrated activation of muscles from both lower extremities.

Reactive balance scores and EMG data were recorded during the experimental task derived from the third day to control for the known impact of any learning effect on participants’ performances (17). Thus, all participants had equal chance to engage in the tests fully familiarized. The second trial on the third day was utilized to allow participants to undergo one exposure trial and formed the data set for this study.

Reactive balance scores were obtained from PROPRIO’s standardized test (Dynamic Movement Analysis^®^, DMA). The DMA score, representative of participants’ reactive balance control performance, is calculated based on the sum of the transmitter’s movement in all directions (anterior–posterior, medial-lateral, vertical). When participants cannot complete the full test (120 s), the software generates an adjusted score by adding 12 points for every second remaining in the test. Of note, given the software’s method of scoring, a participant may exhibit a higher score based on excessive movement during the trial even though the test was completed in full compared with a participant who could not complete the entire trial but exhibited lesser body movement. The minimum and maximum DMA scores are 0 and 1,440 points, respectively, with lower scores indicating better balance.

Direct Current (DC) bias and baseline noise was adjusted prior to collecting EMG data. Acquired EMG data were then digitally filtered (60 Hz notch; 10 Hz high-pass and 350 Hz low-pass Butterworth), full-wave rectified, and integrated over 0.01 s intervals. Processed EMG data were normalized to MMT, expressed as percent of the maximum recorded within a 0.5 s moving window average during the MMT. Intensities were normalized and reported as percent of maximal isometric MMT (% MMT). Only the EMG signals that exceeded the amplitude of 5% MMT were considered significant, defining the onsets and cessations of the EMG envelopes (27, 28). Envelopes of EMG separated by short duration gaps (<0.5 s) were combined into larger packets for analysis (27, 28). A participant’s peak EMG activity during each of the 10 s epochs within the 120 s trial was expressed as percent of the participant’s maximum MMT (% MMT) for the eight muscles studied (i.e., vastus lateralis, medial hamstrings, tibialis anterior, medial gastrocnemius, bilaterally). Then, for each participant, peak EMG values across all 8 muscles were averaged to represent the average lower extremity *composite EMG activity*. Also, to visually represent muscle activity across each epoch of the balance test, we averaged the composite scores from each participant for each group.

### Statistical analyses

To detect differences between the TBI and Control groups on functional measures (BBS, DGI, lower extremity composite muscle strength, and DMA), independent samples *t*-test were performed. When data did not meet normality assumptions (Kolmogorov–Smirnov test), Mann–Whitney U Test was used to detect between-group differences (level of significance set at 0.05).

To investigate control of lower extremity muscle activity between individuals with TBI and Controls during the reactive balance test, we first compared composite EMG activity between groups across each 10 s epoch of the 120 s Propriotest^®^ with Mann–Whitney U Test. A Bonferroni correction was applied to adjust for the increased risk of a type one error given that multiple comparisons within a single family of variables were being performed (i.e., 12 epochs of EMG activity) (29). Level of significance was set at 0.05/12 = 0.004. For this multiple pair-wise comparisons, we selected to not apply a full factorial design as the statistical treatment since an incomplete TBI data set towards the end of the balance test would result in listwise deletion (i.e., participant’s entire data set excluded from statistical comparison). Cohen’s d Effect size was also included to display the magnitude of differences between groups based on established parameters (0.2 = small, 0.5 = medium, ≥0.8 = large) (30). Following, DMA scores were correlated (Spearman’s rank correlation coefficient, *ρ*) with the composite EMG activity, with level of significance set at 0.05.

For our exploratory analysis within the TBI group, descriptive statistics demonstrated muscle activity patterns across each 10 s epoch of the 120 s Propriotest^®^ followed by Spearman rank correlation between DMA scores and functional measures separately for those with unilateral and bilateral lower extremity involvement. All statistical treatments were performed in SigmaPlot 11.0 (Systat Software, Inc.) and SPSS (version 29.0.0, IBM Statistics) for the correlation analysis.

## Results

### Functional measures

**Figure 2:**
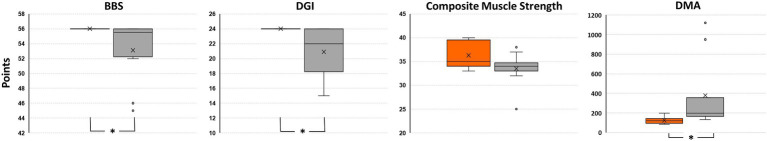
Comparison of Berg Balance Scale (BBS), Dynamic Gait Index (DGI), lower extremity composite muscle strength, and Dynamic Movement Analysis (DMA) scores between Control group (orange box to the left of each comparison) and TBI group (gray box). Asterisk indicates significant difference between groups (Mann–Whitney U Test).

Functional outcomes for both Control and TBI groups can be seen on Figure 2. The BBS scores were higher (*p* = 0.015) for the Control group (mean ± SD of 56 ± 0, median 56) compared to the TBI group (53.1 ± 4.3, median 55.5, range 45–56 points). Similarly, higher (*p* = 0.006) DGI scores were observed for the Control group, in which all participants achieved the maximum score (24 ± 0, median 24) compared to the TBI group (20.9 ± 3.4, median 22, range 15–24 points). Lower extremity composite muscle strength measures did not differ significantly (*p* = 0.068) between the Control (36.3 ± 2.9, median 35, range 33–40 points) and TBI (33.5 ± 3.5, median 34, range 25–38 points) groups. Lastly, lower (*p* = 0.001) reactive balance scores (DMA) were observed for the Control group (124.2 ± 36.1, median 117.5, range 85–196 points) compared with the TBI group (378.5 ± 357.2, median 197.5, range 133–1,120 points).

### Reactive balance performance and lower extremity composite EMG activity

**Figure 3:**
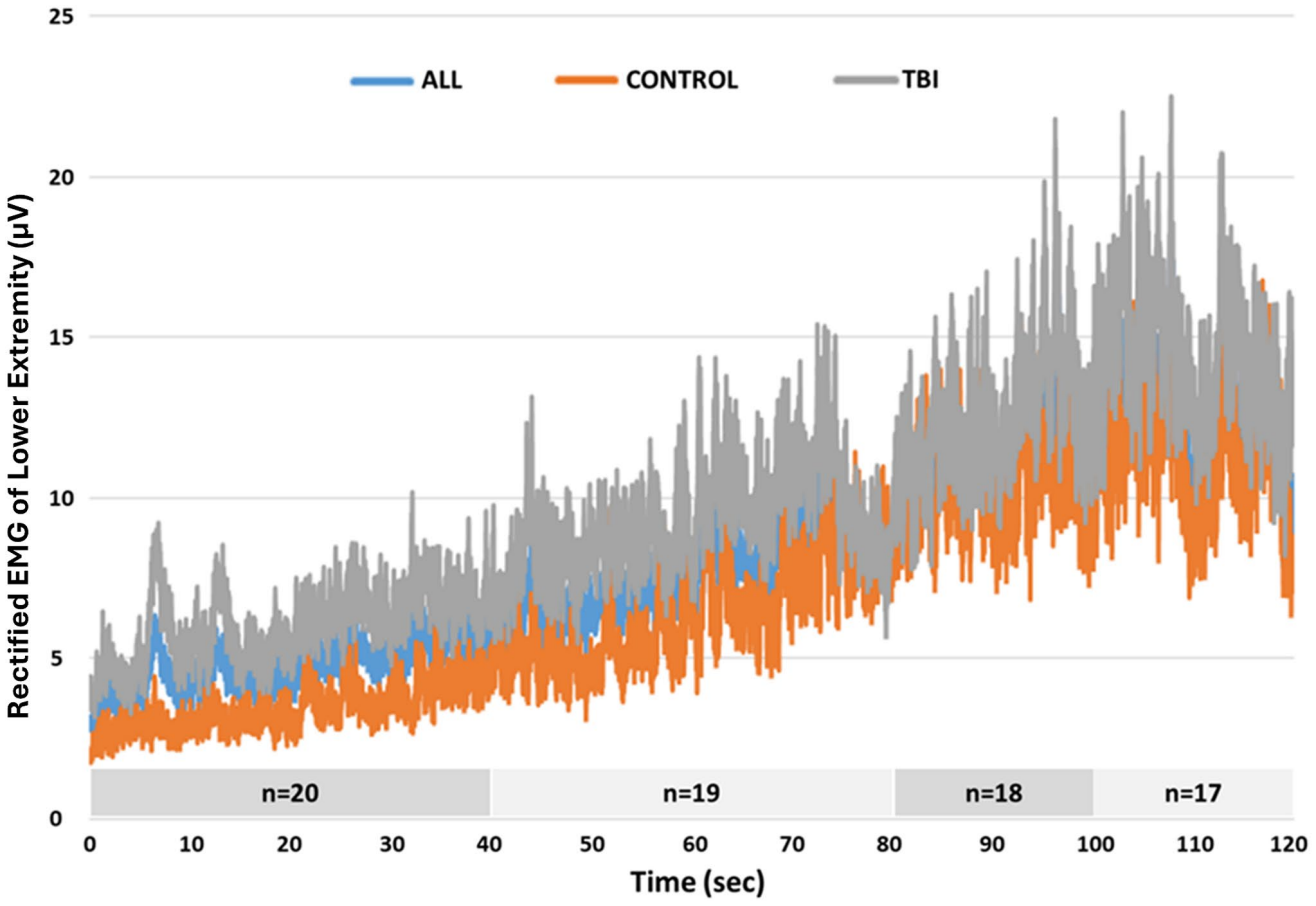
Average rectified EMG activity of lower extremity muscles across the entire 120 s Propriotest^®^.

**Figure 4:**
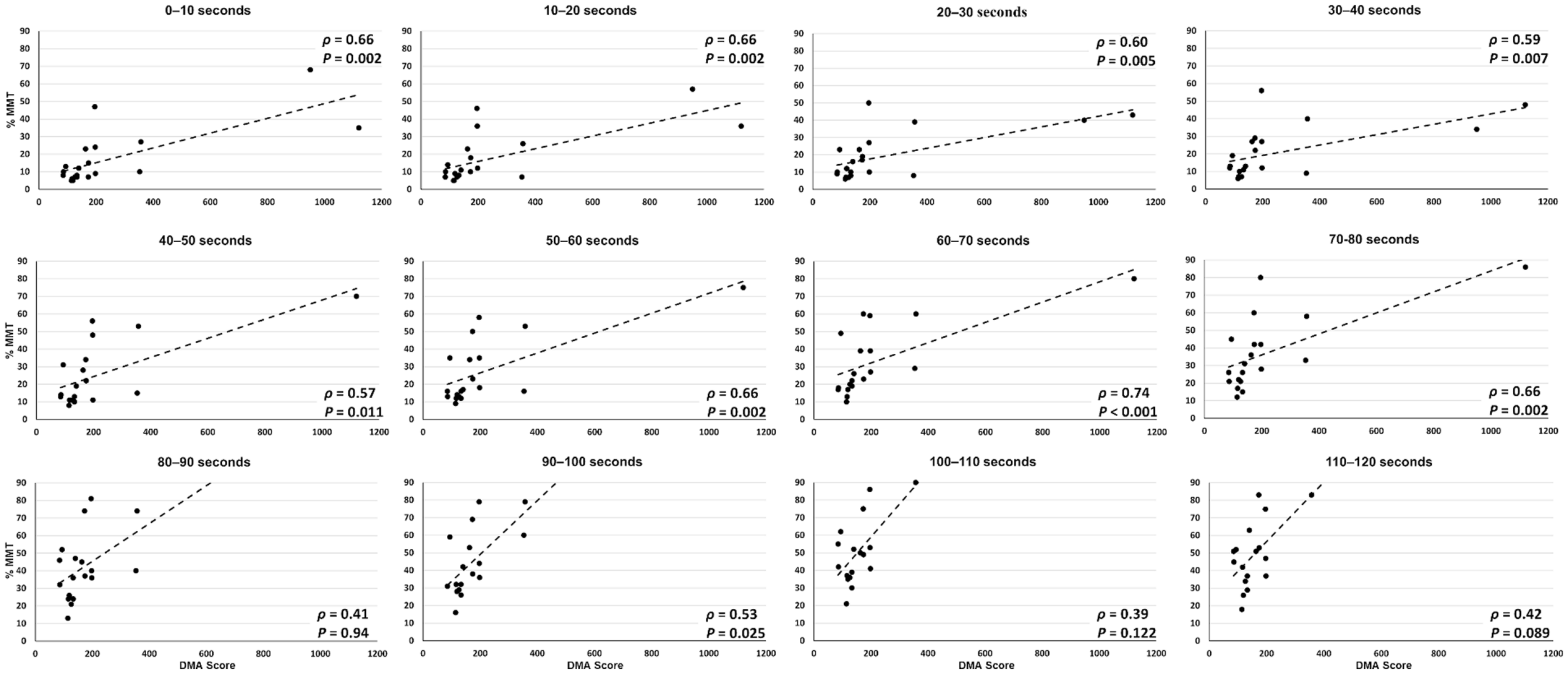
Correlation between composite EMG activity (y axis) and DMA scores (x axis) across all 10 s epochs of the 120 s Propriotest^®^.

Three participants with TBI (participants 1, 3, and 8) were not able to complete the entire 120 s test, losing balance before test completion. Overall, greater lower extremity muscle activity was observed during the reactive balance test for those with poorer DMA scores (Figure 3). However, when contrasting composite EMG activity between groups, significant differences were not observed (Table 2). Greater composite EMG activity correlated significantly with higher DMA scores for all participants combined across all but three 10 s windows (80–90 s; 100–110 s; 110–120 s) of the reactive balance test (Figure 4).

**Table 2:** Composite EMG activity at each 10 s epochs of the reactive balance test Propriotest^®^.

	Control		Traumatic brain injury		*p*	Effect size d
Test epochs	Mean ± SD, Median	Inter-quartile range (Q1–Q3)	Mean ± SD, Median	Inter-quartile range (Q1–Q3)		
0–10	11.5 ± 12.7, 7	5.7–10.7	23.1 ± 18.1, 19	9.7–29	0.01	0.7
10–20	12.1 ± 12.2, 8.5	6.5–11	23.4 ± 15.9, 20.5	10.2–36	0.03	0.8
20–30	15.1 ± 13.3, 10	7–18.5	23.3 ± 13.5, 21	9.5–39.2	0.13	0.6
30–40	17 ± 15.3, 11.5	7–21.5	24.3 ± 13.4, 24.5	11.7–35.5	0.13	0.5
40–50	20.2 ± 15.4, 13	11–31.7	30.7 ± 21.3, 22	13–50.5	0.25	0.6
50–60	23.6 ± 17.6, 15	12.7–38.7	31.4 ± 20.8, 23	16.5–44	0.18	0.4
60–70	28.5 ± 19.4, 19	16–51.5	38 ± 19.9, 29	24.5–49.5	0.09	0.5
70–80	33 ± 21.8, 24	20–48.7	41.2 ± 20.5, 36	29.5–50	0.21	0.4
80–90	40.5 ± 22.7, 34	23.2–57.5	42.9 ± 14.4, 40	36.2–46.5	0.53	0.1
90–100	42.5 ± 20.6, 32	28.7–61.5	47.3 ± 16.5, 43	36.5–58.2	0.42	0.3
100–110	48.8 ± 20.3, 40.5	35.7–65.2	52.1 ± 18.5, 50	41–53	0.66	0.2
110–120	46.3 ± 20.3, 43.5	32–57.7	51.9 ± 17.6, 51	37–63	0.43	0.3

### Exploratory analysis

The DMA scores and lower extremity composite EMG activity across the 120 s test for our cohort of individuals with TBI can be seen on Table 3.

**Table 3:** DMA scores and lower extremity composite EMG activity (% MMT) for individuals with TBI separated by unilateral and bilateral lower extremity residual involvement and ordered increasingly by DMA scores.

Participant	Side involvement	DMA	10 s	20 s	30 s	40 s	50 s	60 s	70 s	80 s	90 s	100 s	110 s	120 s
7	L	133	8	8	8	11	10	12	19	15	24	26	30	29
4	R	140	12	11	16	13	19	17	26	31	47	42	52	63
9	L	163	23	23	23	27	28	34	39	36	45	53	50	51
10	L	174	15	18	19	22	22	23	23	42	37	38	49	53
5	L	197	24	36	27	27	48	35	39	42	40	44	53	47
8	L	1,120	35	36	43	48	70	75	80	86				
6	ND/Bilat	198	9	12	10	12	11	18	27	28	36	36	41	37
1	Bilat	353	10	7	8	9	15	16	29	33	40	60		
2	Bilat	357	27	26	39	40	53	53	60	58	74	79	90	83
3	Bilat	950	68	57	40	34								

Bilat, bilateral; DMA, Dynamic Movement Analysis; L, left; ND, not determined based on Manual Muscle Test and Ashworth Scale; R, right. Note empty cells (participants 1, 3, 8) indicate individual lost balance and did not complete the entire test.

For the separate sub-analysis of lower extremity involvement (Table 4), while DMA scores were significantly correlated with the clinical functional measures BBS and DGI for the individuals with unilateral lower extremity involvement (*n* = 6), this correlation was only observed with BBS for those with bilateral involvement (*n* = 4).

**Table 4:** Exploratory correlation between reactive balance control (DMA) scores and clinical functional measures.

Participants with unilateral lower extremity involvement (*n* = 6)						
*Spearman correlation*		DMA	BBS	DGI	MMT	LOC
*ρ*	BBS	−0.845				
*P*		0.034				
*ρ*	DGI	−0.845	1.000			
*P*		0.034	<0.001			
*ρ*	Sum_MMT	−0.324	0.435	0.435		
*P*		0.531	0.388	0.388		
*ρ*	LOC	0.486	−0.845	−0.845	−0.294	
*P*		0.329	0.034	0.034	0.571	
*ρ*	Years post injury	0.174	−0.600	−0.600	−0.746	0.754
*P*		0.742	0.208	0.208	0.088	0.084

Participants with bilateral lower extremity involvement (*n* = 4)						
*Spearman correlation*		DMA	BBS	DGI	MMT	LOC
*ρ*	BBS	−1.000				
*P*		<0.001				
*ρ*	DGI	−0.800	0.800			
*P*		0.200	0.200			
*ρ*	Sum_MMT	−0.400	0.400	0.800		
*P*		0.600	0.600	0.200		
*ρ*	LOC	0.400	−0.400	−0.200	−0.400	
*P*		0.600	0.600	0.800	0.600	
*ρ*	Years post injury	−0.400	0.400	0.800	1.000	−0.400
*P*		0.600	0.600	0.200	<0.001	0.600

BBS, Berg Balance Scale; DGI, Dynamic Gait Index; DMA, Dynamic Movement Analysis; LOC, loss of consciousness; MMT, manual muscle test.

## Discussion

Reactive balance recovery is crucial for independence in daily ambulatory activities. The impact of severe brain injuries can alter one’s capacity to respond appropriately to external perturbations even several years post-injury (11, 17). Although abnormal muscle activity has been associated with chronic gait deficits following TBI (4), studies to date have not investigated the reactive muscle activity underlying the control of standing posture during dynamic conditions. Our findings suggest that unexpected perturbations, even when small, generate high amplitude lower extremity muscle activity for those with chronic severe TBI. This greater relative muscle effort in contrast to matched control individuals is related with poorer control of reactive balance. Obtaining greater knowledge of this abnormal muscle activity pattern during reactive balance tests, although not statistically significant across the entire test used in our study, is the first step towards elucidating factors contributing to reactive balance deficits during upright posture in individuals with TBI.

In support of our hypothesis, greater lower extremity muscle activity was observed for those with poorer reactive balance control, although this finding was not always statistically significant. Although a small overlap of DMA scores can be observed in Figure 2, significantly larger DMA scores (i.e., poorer balance) were documented for those with TBI compared to their peers without disabilities. On average, the greater muscle activity for those with TBI emerged even with the smallest perturbation, as observed during the first 20 s of the test with the largest effect sizes (Table 2). While this greater muscle activity could suggest more forceful recruitment of relatively weaker muscles by the TBI group when attempting to maintain balance, our between group comparison of lower extremity composite muscle strength did not identify significant differences between the two groups. This could in part, have arisen because the regions of relative weakness or strength (e.g., hip, knee, or ankle) may have varied across participants. Alternatively, it is possible that there were not significant differences in strength between groups, but instead the difference arose in the capacity to selectively and effectively recruit the correct muscles needed to respond to the perturbation. Several factors could contribute to abnormal muscle recruitment of muscle fibers (31) by those with TBI during the reactive balance control test, including impaired central motor processes or sensory integration and diminished sensory acuity in those with brain injury compared with control individuals (32). While a strengthening program would not be precluded, the latter interpretation of impaired recruitment patterns suggests that a rehabilitative program targeting safe community integration and mobility outcomes for those with severe TBI could focus on reactive postural control with small perturbations. Since treatment-oriented visual tactics are readily available, e.g., EMG feedback, clinicians could employ such approaches to investigate whether muscle activity patterns change (i.e., decrease) after exposure to controlled, unexpected external perturbations. Given that individuals with TBI exhibit a higher rate of falls in the community compared with other patient populations and individuals without disabilities (17, 33), our findings may offer a practical strategy for helping reduce falls at home and in the community in individuals with chronic severe TBI. Future work should consider sensory and proprioceptive factors, among other variables, in association with the muscle activity pattern presented in our study to further inform rehabilitation plans towards more complex ambulatory tasks for those with chronic severe TBI.

When considering the heterogeneity of our participants, it appears that the severity of residual deficits can impact reactive balance control. Individuals with more severe balance limitations began to progressively be eliminated from the analysis given inability to complete the balance test. For example, the two individuals with TBI who exhibited the highest DMA scores (1,120 and 950) also exhibited the lowest scores on the clinical measures (BBS: 45 and 46; DGI: 15 and 19) with the lowest scores among all participants achieved on the BBS tasks “standing with one foot in front” and “standing on one leg.” This finding suggests that clinical postural control tasks with decreased base of support (whether by tandem or single limb stance) may be related to impaired reactive balance performance. Moreover, at later time epochs of the reactive balance test, average rectified EMG activity of both groups was more alike (Figure 3), and correlations were not significant, suggesting muscle activity patterns similarly to those without disabilities. Furthermore, once the external perturbation increased, muscle activity appeared once again distinct between groups with greater DMA scores correlating significantly with larger muscle activity. This finding also highlights the interaction between severity of and time away from injury when considering motor outcomes post TBI. While motor recovery (e.g., muscle recruitment capacity) is expected to improve as time from injury increases, the severity of the injury (in our case, LOC) can also contribute to current motor capabilities, impacting reactive balance responses.

Although issues with lower extremity muscle strength have been related with gait and balance impairments in adolescents with moderate-to-severe TBI (34), the lower extremity composite muscle strength observed in our study did not differ significantly between groups. Important to note the clear decrease in variability of strength scores demonstrated by our cohort of individuals with chronic severe TBI (i.e., shorter box plot without the two outliers). In contrast, adults from the Control group exhibited a larger but controlled distribution of strength scores (i.e., larger box plot with greater observation of scores within the quartiles). This distribution was expected given the age range of our participants (21–55 years) and the known impact of age alone explaining up to 25% of variance in muscle strength in neurotypical adults (35). Interestingly, the tendency of greater range in strength scores did not impact the ability of the Control individuals to achieve maximum scores when performing both clinical tests of gait (DGI) and balance (BBS). Even the Control individuals whose strength scores fell below the median TBI strength scores (*n* = 5 with ≤34) achieved the maximum clinical tests scores. In contrast, not all adults from the TBI group who scored above the Control’s median strength score (*n* = 3 with ≥35) were able to achieve DGI and BBS ceiling scores. This finding may also reflect the ceiling effect of the clinical tests used in our study. Future work should investigate such findings with a more homogenous cohort of adults with severe TBI and different clinical assessments to highlight the potential strength measures impact on reactive balance performance. Additionally, future research is warranted to identify factors that may be contributing to the distribution in strength scores within and across groups.

Our exploratory analysis suggested that individuals with chronic severe TBI with bilateral lower extremity involvement may exhibit poorer control of reactive balance in contrast to those with unilateral involvement. The significant relationship observed between better reactive balance control scores and DGI for those with unilateral but not for those with bilateral involvement may highlight the potential ability of independent community integration by those with lesser residual deficits post-injury. Moreover, while BBS scores significantly correlated with DMA scores for both groups, the DGI correlated only for the sub-group with unilateral involvement. This finding may also emphasize the ability of reactive balance control scores detecting those with ambulatory capacity to modify dynamic balance requirements in the presence of external perturbatory stimuli. However, such findings were exploratory and cannot be extrapolated given the limited sample size per sub-group. Future work should explore this investigation to corroborate findings.

### Limitations

A limitation of our work involves the study design, which involves a secondary analysis of an already completed project. Given Institutional Review Board-related activities had been closed when the writings of this study started, changes in recruitment/testing protocol could not be amended. Description of injury location (e.g., images) and information regarding other non-pyramidal systems involvement were not collected in the primary project, limiting further classifications and clinical sysndromes following TBI. In addition, *a priori* sample size estimation could not be performed. Thus, to optimize the rigor of future studies, future work could utilize our study findings and methodology as baseline for a robust protocol design. Follow-up studies with appropriately larger cohorts could confirm whether between-group differences in lower extremity composite EMG activity exist and are maintained across the entire reactive balance test. Furthermore, if future work detects no differences at later time epochs of the test, it should highlight the importance of focusing clinical care on the control of small, unexpected external perturbations as crucial for safe integration in community ambulation for individuals with chronic severe TBI. Given the study implemented a secondary analysis on a published, limited data set, the analysis of an important muscle group responsible for balance control in mediolateral plane was not present in this study. The hip abductors should be considered in future studies to clarify their contribution to the control of reactive postural control.

Another limitation involves the reactive balance test utilized. Although computerized dynamic posturography is considered a dynamic testing condition, participants attempt to maintain upright posture as external perturbations are delivered. The constraints of this experimental task make this evaluation not as dynamic as observed during ambulatory tasks in which velocity (both direction and magnitude) is also a factor to be controlled, especially during tasks that require deceleration such as walking (36) or running (9, 10, 37) termination. Future work should consider more dynamic conditions to elucidate control strategies based on muscle activity patterns to maintain postural stability as it pertains to independence in community ambulatory activities.

## Conclusion

Considering our participants with chronic severe TBI, a tendency for greater lower extremity muscle activity in comparison with those without disabilities was related with poorer reactive balance performance, especially during the initial phase of the reactive balance test. Even a small external perturbation, as observed during the initial seconds of the test, was enough to elicit higher amplitude of muscle activity for those with chronic severe TBI. This pattern, although suggestive of abnormal muscle recruitment, did not consistently reach significant differences when compared to individuals without disabilities. Our findings suggest that even after achieving independent ambulation and community integration, adults with TBI may exhibit altered neuromuscular responses during reactive balance control, particularly early in the perturbation.”

## Data Availability

The datasets presented in this article are not readily available because of ethical and privacy restrictions. Components of the datasets may be made accessible to qualified investigators with the appropriate ethical approvals and data use agreements upon request. Requests to access the datasets should be directed to the corresponding author GMC.
